# Efficacy of Contrast‐Enhanced Endoscopic Ultrasound in the Diagnosis of Gallbladder Tumor: A Retrospective Multicenter Cohort Study

**DOI:** 10.1002/jhbp.70069

**Published:** 2026-01-23

**Authors:** Kensaku Yoshida, Akinori Maruta, Shinya Uemura, Keisuke Iwata, Shogo Shimizu, Tsuyoshi Mukai, Takuji Iwashita, Masahito Shimizu

**Affiliations:** ^1^ Department of Gastroenterology Gifu Prefectural General Medical Center Gifu Japan; ^2^ First Department of Internal Medicine Gifu University Hospital Gifu Japan; ^3^ Department of Gastroenterology Gifu Municipal Hospital Gifu Japan; ^4^ Matsunami General Hospital Gifu Japan; ^5^ Shiga University of Medical Science Hospital Otsu Shiga Japan

**Keywords:** contrast‐enhanced endoscopic ultrasonography, gallbladder wall layer structure, perflubutane

## Abstract

**Background and Aims:**

Contrast‐enhanced endoscopic ultrasonography (CE‐EUS) provides clearer visualization of wall layer structures in gallbladder nodules than B‐mode EUS. This study aimed to compare the diagnostic performance of B‐mode EUS and CE‐EUS in differentiating benign from malignant gallbladder lesions.

**Methods:**

Patients who underwent both B‐mode EUS and CE‐EUS for gallbladder nodules with available pathological diagnoses were retrospectively analyzed. The diagnostic findings of each modality were evaluated by univariate and multivariate analyses.

**Results:**

Eighty‐six patients were included (31 malignant, 55 benign). On B‐mode EUS, a pedunculated shape and presence of a Rokitansky–Aschoff sinus were associated with benignity, whereas diameter ≥ 20 mm and an unclear or disrupted wall layer structure were associated with malignancy. On CE‐EUS, unclear or disrupted wall layer structure was strongly associated with malignancy, whereas enhancement patterns showed only univariate significance. Multivariate analysis identified unclear or disrupted wall layer structure on CE‐EUS and diameter ≥ 20 mm as independent predictors of malignancy. Diagnostic accuracy was significantly higher with CE‐EUS evaluation of wall layer structure (93%) than with B‐mode EUS evaluation of maximum diameter (78%, *p* < 0.001).

**Conclusion:**

CE‐EUS, particularly assessment of wall layer structure, improves differentiation between benign and malignant gallbladder nodules and complements B‐mode EUS.

## Graphical abstract

In this multicenter retrospective study, Yoshida and colleagues showed that contrast‐enhanced endoscopic ultrasonography improved diagnostic accuracy over B‐mode endoscopic ultrasonography for differentiating malignant from benign gallbladder nodules. Evaluation of gallbladder wall layer structure on contrast‐enhanced imaging was the strongest independent predictor of malignancy and improved clinical risk stratification.

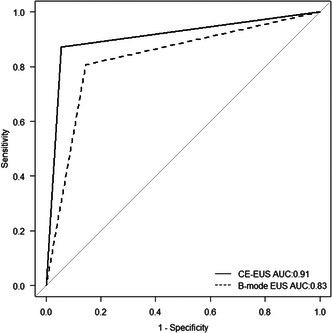

**Abbreviations**
AUC: area under the curve
AUS: abdominal ultrasound
CE‐EUS: contrast‐enhanced endoscopic ultrasonography
EUS: endoscopic ultrasonography
FNA: fine‐needle aspiration
RAS: Rokitansky–Aschoff sinus
ROC: receiver operating characteristic

## 1 Introduction

The diagnosis of benign and malignant gallbladder lesions remains challenging, even with imaging modalities, such as computed tomography, magnetic resonance imaging, and abdominal ultrasound (AUS) [1]. B‐mode endoscopic ultrasonography (EUS) is valuable for detailed observation of gallbladder nodules because it provides higher‐resolution images than those of computed tomography, magnetic resonance imaging, or AUS [1]. The characteristic B‐mode EUS findings of malignant gallbladder nodules include a maximum diameter > 10 mm, a heterogeneous internal echo pattern, a sessile stalk, and loss of wall layer structure [2, 3, 4, 5]. In particular, EUS is useful because evaluation of the layer structure can be difficult with AUS. Reports on B‐mode EUS for gallbladder nodules have described the gallbladder wall as comprising two layers—an inner hypoechoic layer and an outer hyperechoic layer; when the inner hypoechoic layer was unclear or the outer hyperechoic layer was disrupted, the lesion was malignant [6, 7, 8]. Assessment of the gallbladder wall layer structure using B‐mode EUS has been shown to be useful in distinguishing benign from malignant nodules [8].

However, there are limitations to diagnosing benign and malignant gallbladder nodules with B‐mode EUS alone. First, differentiating between biliary sludge and a solid mass can be difficult [9, 10]. Second, accurate evaluation of the gallbladder wall layer structure is challenging in the presence of inflammation or vascular structures [11, 12].

Contrast‐enhanced EUS (CE‐EUS) may aid in differentiating gallbladder nodules that are difficult to characterize using B‐mode EUS alone. Reports have indicated that homogeneous enhancement suggests benignity, whereas heterogeneous enhancement suggests malignancy [13, 14]. In another study, Imazu et al. examined 36 cases of gallbladder wall thickening with both B‐mode EUS and CE‐EUS and reported that wall thickness ≥ 12 mm, disrupted wall layer structure, and inhomogeneous contrast distribution were associated with malignancy [15]. Moreover, evaluation of the inner hypoechoic and outer hyperechoic gallbladder wall layers using CE‐EUS may improve the diagnostic accuracy of B‐mode EUS in differentiating benign from malignant nodules. According to these reports, B‐mode EUS with CE‐EUS is expected to be more accurate than B‐mode EUS alone for diagnosing malignant gallbladder nodules.

To date, however, no study has directly compared B‐mode EUS and CE‐EUS in the diagnosis of benign and malignant gallbladder nodules. This study aimed to compare the usefulness of B‐mode EUS and CE‐EUS for this purpose.

## 2 Methods

### 2.1 Patient Selection

This retrospective cohort study was conducted at three tertiary care centers between April 2012 and December 2022. Patients who underwent CE‐EUS for gallbladder nodules were included in this study. The exclusion criterion was the lack of a final pathological diagnosis obtained through surgery or needle biopsy. This study was conducted in accordance with the principles of the Declaration of Helsinki. This multicenter retrospective study was approved by the institutional review board of Gifu University Hospital (Approval No. 2022‐298). The requirement for informed consent was waived due to the retrospective nature of the study.

### 2.2 EUS Procedure

EUS was performed under moderate sedation with midazolam and pentazocine in all patients. A convex‐type echoendoscope (GF‐UCT260; Olympus, Tokyo, Japan), a radial array echoendoscope (GF‐UE260; Olympus, Tokyo, Japan), and US systems (ProSound F75 or ProSound Alpha 10; FUJIFILM, Tokyo, Japan) were used for all procedures. First, B‐mode EUS was performed to evaluate the characteristics of gallbladder nodules. The transmitting frequency and mechanical index were set at 5 MHz and 0.2, respectively, for CE‐EUS in extended pure harmonic detection mode. A bolus infusion of 0.015 mL/kg body weight of the contrast agent, perfluorobutane with a median diameter of 2–3 μm (Sonazoid: Daiichi‐Sankyo, Tokyo, Japan), was administered through a peripheral vein. CE‐EUS of the gallbladder nodule was performed for at least 60 s after observing the gallbladder using B‐mode EUS. The images acquired by CE‐EUS were evaluated 30–45 s after contrast agent administration. All B‐mode EUS and CE‐EUS examinations recorded in AVI format were independently evaluated by two board‐certified endoscopists with more than 10 years of EUS experience and over 500 gallbladder EUS examinations. Both reviewers were also certified instructors of the Japan Biliary Association. After the procedure, the patients were observed in the endoscopy room for at least 2 h to monitor for possible adverse events.

### 2.3 Image Analysis on B‐Mode EUS

The following findings were evaluated in B‐mode EUS: maximum diameter, shape (sessile or pedunculated), Rokitansky–Aschoff sinus (RAS) (presence or absence), internal echo pattern (homogeneous or heterogeneous), internal echogenicity (hyperechoic or hypoechoic), and wall layer structure. The presence of two layers on the gallbladder wall, an internal hypoechoic layer and outer hyperechoic layer, was evaluated using B‐mode EUS. The hypoechoic layer consists of the mucosa, muscularis propria, and subserosa, and the hyperechoic layer corresponds to the serosa [7, 16]. Regarding wall layer structure, the internal hypoechoic layer was classified as clear or unclear, and the outer hyperechoic layer was classified as clear or disrupted, consistent with previous studies (Figure S1) [7, 17]. Both the inner hypoechoic layer and the outer hyperechoic layer were evaluated independently in all cases. In our cohort, lesions with an unclear inner hypoechoic layer always retained a clear outer hyperechoic layer, whereas lesions with a disrupted outer hyperechoic layer consistently showed an unclear inner layer. Thus, disrupted outer hyperechoic layer invariably coexisted with an unclear inner hypoechoic layer, while an unclear inner hypoechoic layer did not necessarily indicate a disrupted outer hyperechoic layer.

### 2.4 Image Analysis on CE‐EUS

CE‐EUS was used to evaluate the wall layer structure and enhancement pattern of the gallbladder nodules. Classification of the wall layer structure of the gallbladder nodules on CE‐EUS was similar to that on B‐mode EUS (Figure S2). The enhancement pattern of the gallbladder nodules on CE‐EUS was classified into three patterns: homogeneous enhancement, heterogeneous enhancement, and no enhancement, as described in previous studies (Figure S3) [6, 13, 14, 18, 19].

### 2.5 Interobserver Agreement

The κ coefficient was used to assess reproducibility between assessments by the two physicians. Cohen's κ values were calculated for each B‐mode EUS finding (wall layer structure, presence of Rokitansky–Aschoff sinus, echo texture, echogenicity, and shape) and each CE‐EUS finding (wall layer structure and enhancement pattern). Discrepancies between the two physicians were resolved by consensus. Cohen's κ values were interpreted according to the criteria proposed by Landis and Koch: poor (< 0.20), fair (0.21–0.40), moderate (0.41–0.60), substantial (0.61–0.80), and almost perfect (> 0.80).

### 2.6 Final Diagnosis and Definitions

The final diagnosis was made based on the pathological examination of specimens obtained by surgery. If a surgical sample was not available, specimens obtained by fine‐needle aspiration (FNA) were used for the final diagnosis. EUS‐FNA was not routinely performed for gallbladder nodules in this study. It was limited to a small number of selected patients, including four cases of locally advanced unresectable gallbladder cancer and two benign cases with high surgical risk, in whom histological confirmation was clinically required. In all cases, EUS‐FNA was performed using a 22‐gauge needle via the duodenal bulb. No procedure‐related adverse events, including bile leakage or tumor seeding, were observed. Malignant gallbladder nodules were defined as adenocarcinoma, adenosquamous carcinoma, carcinosarcoma, metastatic tumor, neuroendocrine tumor, or malignant lymphoma. Benign gallbladder nodules were defined as biliary sludge, cholesterol polyps, inflammatory polyps, chronic cholecystitis, adenomyomatosis, adenoma, or xanthogranulomatous cholecystitis. In cases where the diagnosis was benign on EUS‐FNA, it was confirmed with an appropriate clinical course, including imaging analysis, for at least 1 year of follow‐up.

### 2.7 Study Outcomes and Statistical Analysis

The primary outcome was findings suggestive of gallbladder nodule malignancy on B‐mode and CE‐EUS. The secondary outcome was evaluation of the diagnostic capability of B‐mode EUS and CE‐EUS in differentiating between malignant and benign gallbladder nodules. Continuous variables were compared using the Mann–Whitney *U*‐test. The receiver operating characteristic (ROC) analysis was performed to evaluate the diagnostic performance of representative B‐mode EUS findings and CE‐EUS findings for predicting malignancy. Fisher's exact test or the chi‐square test was used for categorical variables. All B‐mode and CE‐EUS variables were first evaluated using univariate logistic regression. Variables with a *p*‐value of < 0.05 were considered for inclusion in the multivariate model. Variables causing complete separation and variables showing strong collinearity were excluded. The final multivariate logistic regression model was constructed using the forced‐entry method. A *p*‐value of < 0.05 with a two‐sided test was considered statistically significant. All statistical analyses were performed using JMP software, version 11.2 (SAS Institute, Cary, NC, USA).

## 3 Results

### 3.1 Patient Characteristics

Database analysis identified 163 patients who underwent CE‐EUS for gallbladder nodules. However, 77 patients were excluded because of the lack of a pathological diagnosis. Finally, 86 patients, including 38 men with a median age of 68 years (range, 35–92), were enrolled in this study (Figure S4). The locations of the gallbladder nodules were as follows: neck (*n* = 7), body (*n* = 52), and fundus (*n* = 27). The median follow‐up period was 1223 days (range, 93–4160). The diagnosis was confirmed by surgical resection in 80 patients and fine‐needle aspiration in six patients. The pathological diagnoses of the 86 patients included gallbladder adenocarcinoma (*n* = 31), adenomyomatosis (*n* = 17), chronic cholecystitis (*n* = 13), biliary sludge (*n* = 10), cholesterol polyps (*n* = 8), inflammatory polyps (*n* = 2), adenomas (*n* = 3), and xanthogranulomatous cholecystitis (XGC) (*n* = 2). The κ‐coefficient between the two physicians for differentiating benign from malignant gallbladder nodules on B‐mode and CE‐EUS was 0.79. Patient characteristics are shown in Table 1.

**TABLE 1:** Characteristics of patients.

	*N* = 86		*p*
	Malignant (*N* = 31)	Benign (*N* = 55)	
Age, median (range)	74 (54–92)	65 (35–88)	0.001
Sex, *n* (%)			
Men	14 (45)	24 (44)	0.891
Women	17 (55)	31 (56)	
Location, *n* (%)			
Neck	2 (7)	5 (9)	0.539
Body	17 (55)	35 (64)	
Fundus	12 (38)	15 (27)	
Final diagnosis, *n* (%)			
Surgery	27 (87)	53 (96)	0.114
EUS‐FNA	4 (13)	2 (4)	
Histopathological findings, *n* (%)			
Adenomyomatosis		17 (30)	
Chronic cholecystitis		13 (24)	
Biliary sludge		10 (18)	
Cholesterol polyp		8 (14)	
Inflammatory polyp		2 (4)	
Adenoma		3 (6)	
Xanthogranulomatous cholecystitis		2 (4)	
Adenocarcinoma	31 (100)		

Abbreviation: EUS‐FNA, endoscopic ultrasonography guided fine‐needle aspiration.

### 3.2 Results of B‐Mode EUS Findings

The median maximum diameter of the gallbladder nodules was 16 mm (range, 5–63 mm). Although 16 mm represented the median diameter in our cohort, ROC curve analysis identified 20 mm as the optimal cutoff value for predicting malignancy (AUC: 0.88). The updated diagnostic performance based on this ROC curve analysis was presented in Figure S5. A maximum diameter of ≥ 20 mm was observed in 25 of 31 (81%) malignant gallbladder nodules and in 8 of 55 (15%) benign nodules (*p* < 0.001). RAS was present in 14 of 55 (25%) benign nodules and in zero of 31 (0%) malignant nodules (*p* < 0.001). A pedunculated shape was observed in 13 of 55 (24%) benign nodules and in zero of 31 (0%) malignant nodules (*p* < 0.001). The wall layer structure was unclear or disrupted in 25 of 31 (81%) malignant nodules and in 15 of 55 (27%) benign nodules (*p* < 0.001). In univariate analysis for differentiating benign from malignant nodules on B‐mode EUS, a pedunculated shape and the presence of RAS were statistically significant indicators of benignity, whereas a maximum diameter of ≥ 20 mm and an unclear or disrupted wall layer structure were statistically significant indicators of malignancy. The results of B‐mode EUS are shown in Table 2.

**TABLE 2:** Univariate analysis of the benign and malignant gallbladder nodules in B‐mode EUS and CE‐EUS.

Modality	Categorical variables		Malignant	Benign	*p*
			(*N* = 31)	(*N* = 55)	
B‐mode EUS	Wall layer structure, *n* (%)	Clear	6 (19)	40 (73)	< 0.001
		Unclear or disrupted	25 (81)	15 (27)	
	RAS, *n* (%)	Yes	0	14 (25)	< 0.001
		No	31 (100)	41 (75)	
	Echo texture, *n* (%)	Homogeneous	22 (71)	45 (82)	0.249
		Heterogeneous	9 (29)	10 (18)	
	Echo genicity, *n* (%)	Hyperechoic	26 (84)	50 (91)	0.337
		Hypoechoic	5 (16)	5 (9)	
	Shape, (%)	Pedunculated	0	13 (24)	< 0.001
		Sessile	31 (100)	42 (76)	
	Maximum diameter, (%)	< 20 mm	6 (19)	47 (85)	< 0.001
		≥ 20 mm	25 (81)	8 (15)	
CE‐EUS	Enhancement pattern, (%)	Homogeneous or non	13 (42)	54 (98)	< 0.001
		Heterogeneous	18 (58)	1 (2)	
	Wall layer structure, (%)	Clear	4 (13)	52 (95)	< 0.001
		Unclear or disrupted	27 (87)	3 (5)	

Abbreviations: CE‐EUS, contrast‐enhanced endoscopic ultrasonography; EUS, endoscopic ultrasonography; RAS, Rokitansky‐Aschoff sinus.

### 3.3 Results of CE‐EUS Findings

**FIGURE 1:**
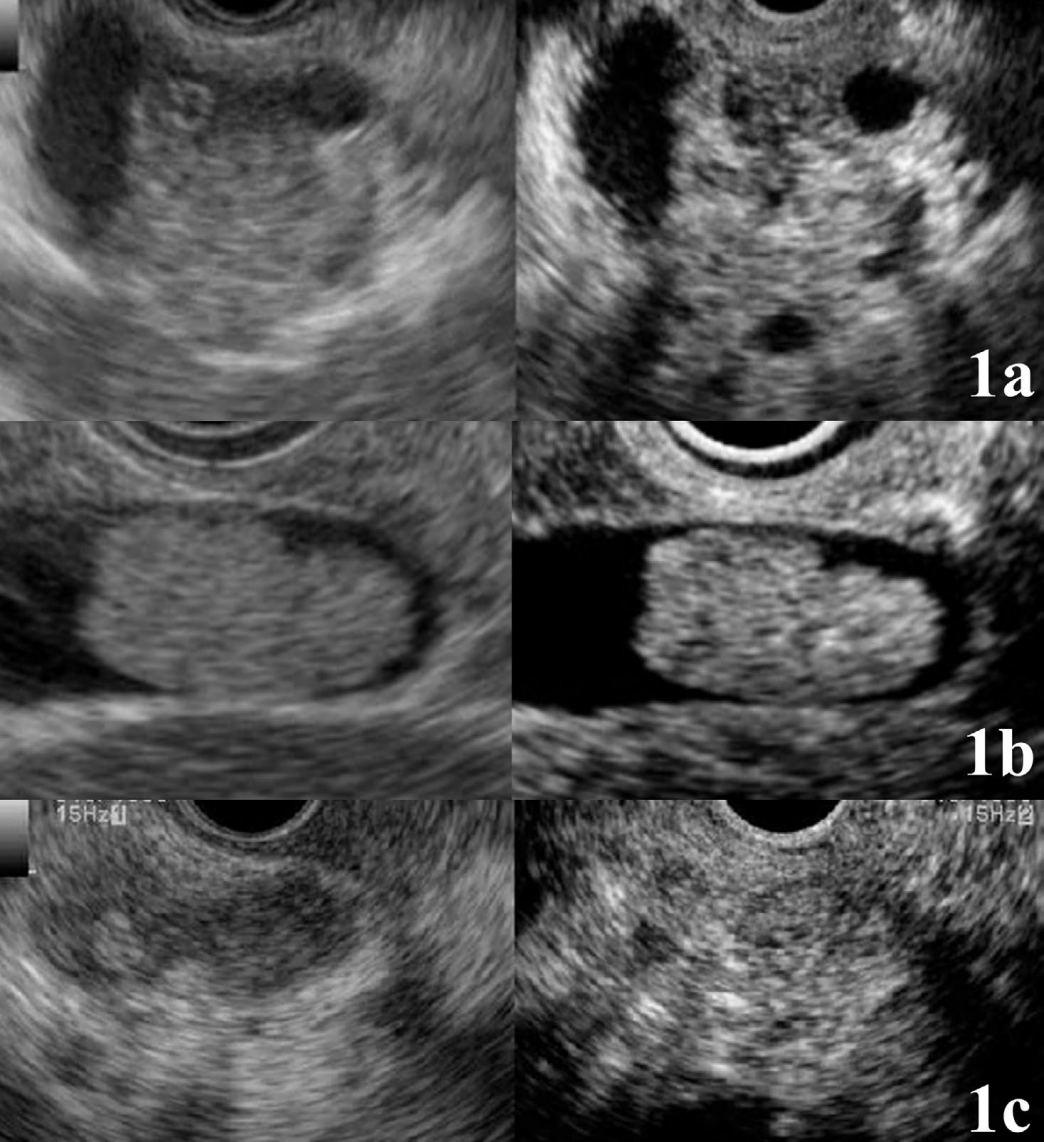
Representative CE‐EUS images showing (a) advanced gallbladder cancer with disrupted wall layer structure (T3), (b) early gallbladder cancer with clear wall layer structure (T1a), and (c) xanthogranulomatous cholecystitis with unclear wall layer structure resulting in a false‐positive interpretation. CE‐EUS, contrast‐enhanced endoscopic ultrasonography.

All gallbladder nodules without enhancement on CE‐EUS were pathologically confirmed as gallbladder sludge. Heterogeneous enhancement was observed in 18 of 31 (58%) malignant nodules and in one of 55 (2%) benign nodules (*p* < 0.001). The wall layer structure was unclear or disrupted in 27 of 31 (87%) malignant nodules and in three of 55 (5%) benign nodules (*p* < 0.001). The results of the enhancement pattern and wall layer structure on CE‐EUS were shown in Table S1. Among the false‐positive cases, one lesion showed a malignant enhancement pattern on CE‐EUS but was ultimately diagnosed as XGC. In contrast, three lesions were falsely interpreted as malignant based on CE‐EUS with wall layer structure findings, including two XGC lesions and one adenomyomatosis, due to unclear or disrupted wall layer structure. These benign inflammatory or hyperplastic conditions represented important mimickers of malignant findings when CE‐EUS was interpreted qualitatively (Figure 1). In univariate analysis of CE‐EUS, an unclear or disrupted wall layer structure and a heterogeneous enhancement pattern were statistically significant indicators of malignancy. The results of univariate analysis using CE‐EUS were shown in Table 2. To further evaluate whether CE‐EUS findings correlated with pathological invasion depth, a stage‐stratified analysis was performed among malignant lesions. Pathological T‐stage information was available for all malignant cases. An unclear or disrupted wall layer structure was present in 20% of Tis and T1a/b lesions, whereas all lesions with invasion ≥ T2 exhibited an unclear or disrupted wall layer structure (*p* < 0.001). With respect to enhancement patterns, none of the Tis or T1a/b lesions demonstrated heterogeneous enhancement (0%), while heterogeneous enhancement was observed in 69% of lesions with invasion ≥ T2 (*p* = 0.002). Wall layer structures and enhancement patterns on CE‐EUS according to depth of invasion in gallbladder cancer were summarized in Table S2. No adverse events related to the endoscopic procedures, including administration of the contrast agent, were observed.

### 3.4 Results of B‐Mode EUS and CE‐EUS

**FIGURE 2:**
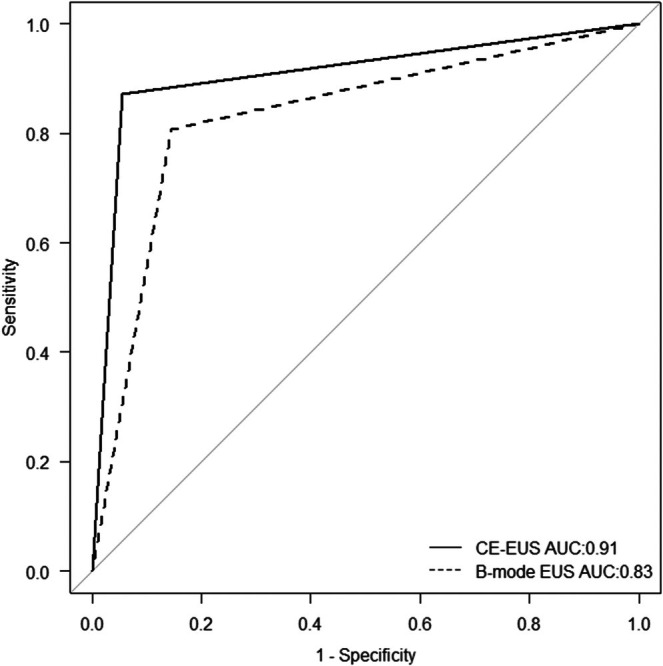
ROC curves comparing B‐mode EUS maximum diameter and CE‐EUS wall layer structure for differentiating benign from malignant gallbladder nodules (AUC 0.83 vs. 0.91). AUC, area under the curve; CE‐EUS, contrast‐enhanced endoscopic ultrasonography; EUS, endoscopic ultrasonography; ROC, receiver operating characteristic.

Because wall layer structure assessed by CE‐EUS demonstrated superior discriminatory ability compared with wall layer structure assessed by B‐mode EUS in ROC analysis (AUC 0.91 vs. 0.77) (Figure S6), the CE‐EUS–based wall layer structure was selected for inclusion in the multivariate model. Multivariate analysis identified a maximum diameter ≥ 20 mm on B‐mode EUS (OR 10.5; *p* = 0.012) and an unclear or disrupted wall layer structure on CE‐EUS (OR 49.9; *p* < 0.001) as independent predictors of malignant gallbladder nodules. In contrast, the enhancement pattern on CE‐EUS was not independently associated with malignancy in the multivariate model (*p* = 0.296) (Table 3). The diagnostic accuracy of CE‐EUS based on wall layer structure (93%) was significantly higher than that of B‐mode EUS based on maximum diameter (78%, *p* < 0.001) (Table 4). To further evaluate diagnostic performance, we compared the discriminatory ability of key individual parameters, namely maximum diameter on B‐mode EUS and wall layer structure on CE‐EUS, using ROC analysis. The maximum diameter assessed by B‐mode EUS showed moderate discriminatory ability, with an AUC of 0.83, whereas wall layer structure evaluated by CE‐EUS demonstrated superior diagnostic performance, yielding an AUC of 0.91. The corresponding ROC curves were presented in Figure 2.

**TABLE 3:** Multivariate analysis of the benign and malignant gallbladder nodules in B‐mode EUS and CE‐EUS.

Modality	Categorical variables		Malignant	Benign	Odds ratio	*p*
B‐mode EUS	Maximum diameter, *n* (%)	< 20 mm	6 (19)	47 (85)	10.5	0.012
		≥ 20 mm	25 (81)	8 (15)		
CE‐EUS	Wall layer structure, *n* (%)	Clear	4 (13)	52 (95)	49.9	< 0.001
		Unclear or disrupted	27 (87)	3 (5)		
	Enhancement pattern, *n* (%)	Homogeneous or non	13 (42)	54 (98)	3.9	0.296
		Heterogeneous	18 (58)	1 (2)		

Abbreviations: CE‐EUS, contrast‐enhanced endoscopic ultrasonography; EUS, endoscopic ultrasonography.

**TABLE 4:** Sensitivity, specificity, and accuracy in B‐mode EUS assessment with maximum diameter and in CE‐EUS assessment with wall layer structure.

Modality	Categorical variables	Sensitivity (%)	Specificity (%)	Accuracy (%)
B‐mode EUS	Maximum diameter	64	91	78
CE‐EUS	Wall layer structure	90	95	93

Abbreviations: CE‐EUS, contrast‐enhanced endoscopic ultrasonography; EUS, endoscopic ultrasonography.

### 3.5 Interobserver Agreement

The overall κ coefficient for differentiating benign from malignant gallbladder nodules was 0.79, which represented the median κ value across all evaluated variables. κ values for individual B‐mode EUS findings ranged from 0.72 to 0.85, indicating substantial to almost perfect agreement. For CE‐EUS findings, κ values ranged from 0.79 to 0.81, also indicating substantial to almost perfect agreement (Table S3).

## 4 Discussion

B‐mode EUS was useful in distinguishing benign from malignant gallbladder nodules by evaluating maximum diameter (*p* < 0.001), shape (*p* < 0.001), presence or absence of RAS (*p* < 0.001), and wall layer structure (*p* < 0.001). CE‐EUS was useful in distinguishing benign from malignant nodules by evaluating contrast pattern (*p* < 0.001) and wall layer structure (*p* < 0.001). Multivariate analysis revealed that a larger maximum diameter on B‐mode EUS and an unclear or disrupted wall layer structure on CE‐EUS were independent risk factors for malignant gallbladder nodules. Among these, evaluation of the wall layer structure using CE‐EUS yielded the highest accuracy rate of 93%.

Regarding B‐mode EUS for malignant features of gallbladder polyps, Park et al. enrolled 689 patients with gallbladder polyps to determine optimal practice guidelines for surgical treatment and follow‐up. They reported that sessile shape and size ≥ 10 mm were risk factors for malignancy [20]. A systematic review, including 53 articles and 6100 cases, reported that the optimal size cutoff for resection was 10 mm and sessile polyps were independent risk factors [21]. European guidelines recommend cholecystectomy in patients with polypoid lesions of the gallbladder measuring ≥ 10 mm, and sessile lesions were considered risk factors for malignancy [22]. In this study, B‐mode EUS revealed that all nodules < 10 mm and all pedunculated nodules were benign. Therefore, the results of the present study are consistent with previous reports.

Homogeneous enhancement suggests benignity, whereas heterogeneous enhancement suggests malignancy in CE‐EUS of gallbladder lesions [6, 13, 14, 15]. However, Kitano et al. reported that, owing to low‐quality evidence, further studies are required to evaluate the role of CE‐EUS in lesion characterization [23]. In the present study, 13 of 57 nodules with homogeneous enhancement were malignant, suggesting that enhancement pattern alone is insufficient to distinguish between benign and malignant lesions. Regarding wall layer structure evaluation with B‐mode EUS, Toyonaga et al. compared 20 cases of gallbladder cancer and reported that the inner hypoechoic layer was clear in T1 lesions, whereas it was unclear or the outer hyperechoic layer was disrupted in lesions deeper than T2 [7]. With CE‐EUS, Imazu et al. evaluated gallbladder wall thickness in 36 cases and reported that thickness ≥ 12 mm was characteristic of malignancy; they also found CE‐EUS superior to B‐mode EUS for evaluating wall layer structure [15]. In the present study, both modalities were useful in differentiating benign from malignant lesions by assessing whether the inner hypoechoic layer was clear or unclear, but CE‐EUS was more accurate [15]. Notably, to our knowledge, no prior report has confirmed that an unclear inner hypoechoic layer is a characteristic of malignancy on CE‐EUS. Thus, performing CE‐EUS in addition to B‐mode EUS is recommended when evaluating gallbladder wall layer structure.

In our cohort, benign lesions mimicking malignancy were observed, indicating limitations of qualitative CE‐EUS assessment. One lesion demonstrated a malignant enhancement pattern despite a final diagnosis of XGC, and three benign lesions (two XGC and one adenomyomatosis) were falsely classified as malignant based on CE‐EUS wall layer structure because their wall layers appeared unclear or disrupted. These findings suggested a limitation of qualitative CE‐EUS assessment, as benign inflammatory or hyperplastic lesions could sometimes resemble malignant features. In the present cohort, benign lesions often demonstrated homogeneous enhancement with relatively delayed washout, whereas malignant lesions tended to show earlier washout. This tendency was consistent with previous studies of contrast‐enhanced ultrasonography of gallbladder lesions, which have reported earlier washout in malignant tumors and relatively delayed washout in benign inflammatory condition [24, 25]. To overcome such limitations of qualitative assessment, quantitative approaches such as time‐intensity curve (TIC) analysis may be useful. TIC parameters, including peak intensity, wash‐in and washout slopes, and time to peak, provide objective indices of microvascular perfusion and have been reported to improve lesion characterization in CE‐EUS [26]. Incorporation of quantitative perfusion analysis focusing on enhancement and washout dynamics may therefore help differentiate benign inflammatory lesions, including XGC or adenomyomatosis, from true malignancy, especially in diagnostically challenging cases. This issue warrants further investigation in future studies.

**FIGURE 3:**
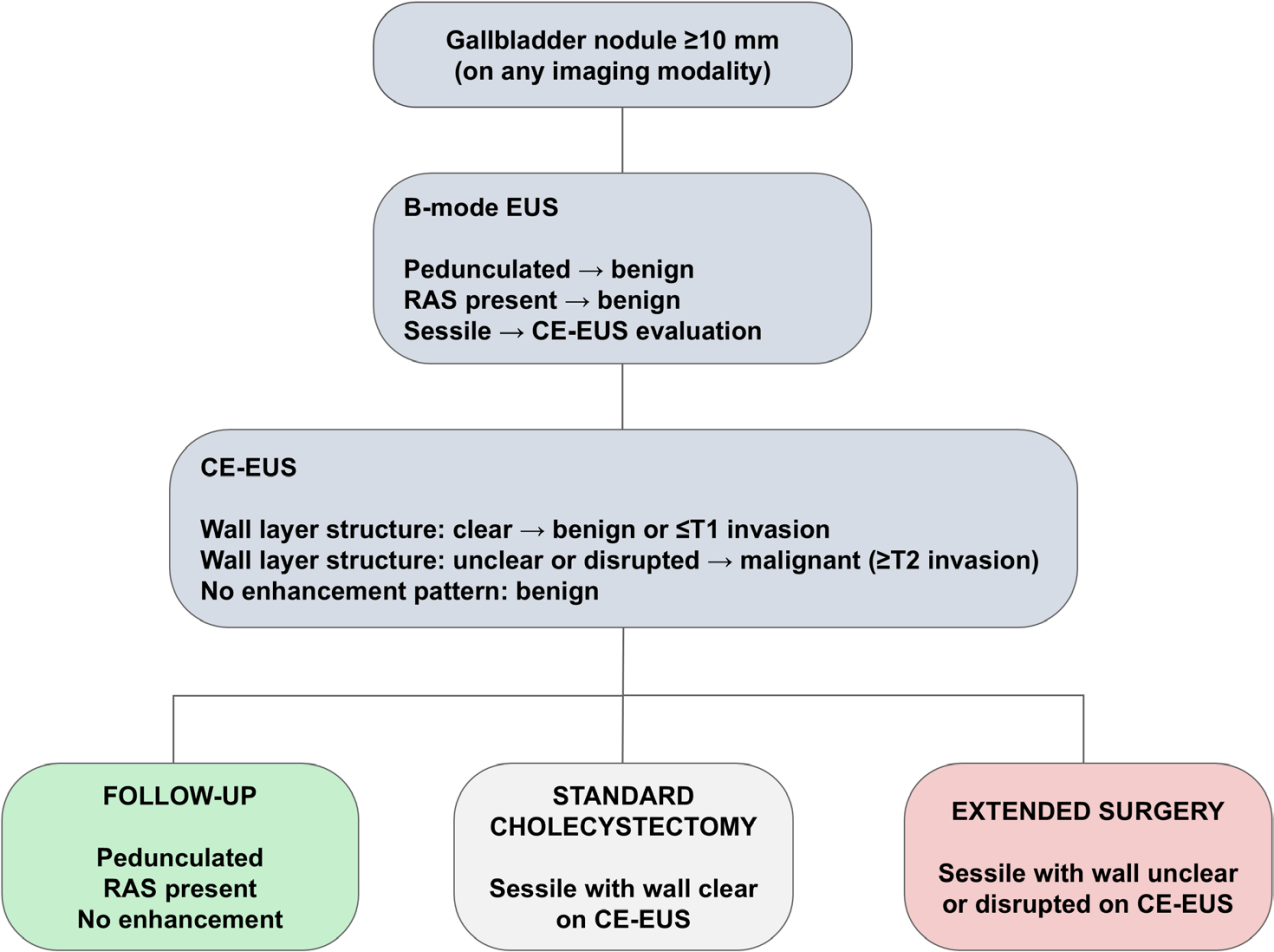
Clinical decision algorithm based on B‐mode EUS and CE‐EUS findings for gallbladder nodules. CE‐EUS, contrast‐enhanced endoscopic ultrasonography; EUS, endoscopic ultrasonography; RAS, Rokitansky–Aschoff sinus.

When focusing on wall layer structure on CE‐EUS in relation to depth of invasion, four malignant nodules exhibited a clear wall layer structure, all of which were limited to Tis or T1a disease. In contrast, all malignant nodules with invasion deeper than T1b demonstrated an unclear or disrupted wall layer structure and were correctly identified using CE‐EUS. These findings suggest that preoperative evaluation of wall layer structure by CE‐EUS may not only aid in differentiating benign and malignant gallbladder nodules but also help identify early‐stage malignancies suitable for reduced‐volume surgery. Nevertheless, XGC remains particularly challenging to distinguish from malignancy using CE‐EUS alone, underscoring the need for further studies. These considerations are summarized in a proposed clinical decision algorithm (Figure 3) that integrates key findings from both B‐mode EUS and CE‐EUS and shows how these results can be applied in clinical practice.

### 4.1 Limitations

This study has limitations. First, the number of patients was relatively small. Second, it was a retrospective study without a control group. Third, some cases were accompanied by inflammation, and the condition of the nodules was not uniform. Fourth, evaluation of wall layer structure and enhancement pattern was performed subjectively by two endoscopists rather than objectively. Fifth, EUS‐guided FNA was performed in only a limited number of selected patients and was not routinely applied, which may have introduced minimal procedural bias in the assessment of diagnostic performance. Larger studies involving more endoscopists are required to clarify the usefulness of CE‐EUS for gallbladder nodules.

In conclusion, the absence of RAS, larger maximum diameter on B‐mode EUS, and unclear or disrupted wall layer structure on CE‐EUS were suggestive of malignant gallbladder nodules in this study. Moreover, CE‐EUS improved the accuracy of distinguishing benign from malignant nodules compared with B‐mode EUS alone. In particular, when B‐mode EUS reveals a sessile gallbladder nodule, CE‐EUS should be performed to differentiate between benign and malignant nodules.

## Author Contributions

K.Y. conducted data abstraction and analysis and drafted the manuscript. A.M. and T.I. supervised the report. All authors have read and approved the manuscript.

## Conflicts of Interest

The authors declare no conflicts of interest.

## Supporting information


**Table S1:** Wall layer structure and enhancement pattern of the benign and malignant gallbladder nodules in CE‐EUS.
**Table S2:** Wall layer structures and enhancement patterns on CE‐EUS according to depth of invasion in gallbladder cancer.
**Table S3:** Interobserver agreement for B‐mode EUS and CE‐EUS findings in gallbladder nodules.

**Figure S1:** Typical B‐mode EUS images demonstrating gallbladder wall layer structure: (a) a benign gallbladder nodule (cholesterol polyp) with a clear internal hypoechoic layer, (b) a malignant gallbladder nodule (adenocarcinoma) with an unclear internal hypoechoic layer, and (c) a malignant gallbladder nodule (adenocarcinoma) with disruption of the outer hyperechoic layer. EUS, endoscopic ultrasonography.
**Figure S2:** Typical CE‐EUS images demonstrating gallbladder wall layer structure: (a) a benign gallbladder nodule (cholesterol polyp) with a clear internal hypoechoic layer, (b) a malignant gallbladder nodule (adenocarcinoma) with an unclear inner hypoechoic layer, and (c) a malignant gallbladder nodule (adenocarcinoma) with disruption of the outer hyperechoic layer. CE‐EUS, contrast‐enhanced endoscopic ultrasonography.
**Figure S3:** Typical CE‐EUS images demonstrating enhancement patterns of gallbladder nodules: (a) a benign gallbladder nodule (adenoma) with homogeneous enhancement, (b) a malignant gallbladder nodule (adenocarcinoma) with heterogeneous enhancement, and (c) a benign gallbladder nodule (gallbladder sludge) showing no enhancement. CE‐EUS, contrast‐enhanced endoscopic ultrasonography.
**Figure S4:** Flowchart of study enrollment. CE‐EUS, contrast‐enhanced endoscopic ultrasonography.
**Figure S5:** Receiver operating characteristic (ROC) curve of the maximum diameter measured by B‐mode EUS for differentiating benign from malignant gallbladder nodules. ROC, Receiver operating characteristic; EUS, endoscopic ultrasonography.
**Figure S6:** ROC curves comparing B‐mode EUS and CE‐EUS wall layer structure for differentiating benign from malignant gallbladder nodules (AUC 0.77 vs. 0.91) ROC, Receiver operating characteristic; EUS, endoscopic ultrasonography; CE‐EUS, contrast‐enhanced endoscopic ultrasonography.

## Data Availability

The data that support the findings of this study are available from the corresponding author upon reasonable request.

## References

[1] T. Azuma , T. Yoshikawa , T. Araida , and K. Takasaki , “Differential Diagnosis of Polypoid Lesions of the Gallbladder by Endoscopic Ultrasonography,” American Journal of Surgery 181, no. 1 (2001): 65–70, 10.1016/S0002-9610(00)00526-2. 11248179

[2] W. B. Choi , S. K. Lee , M. H. Kim , et al., “A New Strategy to Predict the Neoplastic Polyps of the Gallbladder Based on a Scoring System Using EUS,” Gastrointestinal Endoscopy 52, no. 3 (2000): 372–379, 10.1067/mge.2000.108041. 10968853

[3] Y. Sadamoto , S. Oda , M. Tanaka , et al., “A Useful Approach to the Differential Diagnosis of Small Polypoid Lesions of the Gallbladder, Utilizing an Endoscopic Ultrasound Scoring System,” Endoscopy 34, no. 12 (2002): 959–965, 10.1055/s-2002-35859. 12471539

[4] C. Terzi , S. Sökmen , S. Seçkin , L. Albayrak , and M. Uǧurlu , “Polypoid Lesions of the Gallbladder: Report of 100 Cases With Special Reference to Operative Indications,” Surgery 127, no. 6 (2000): 622–627, 10.1067/msy.2000.105870. 10840356

[5] J. H. Kim , J. Y. Lee , J. H. Baek , et al., “High‐Resolution Sonography for Distinguishing Neoplastic Gallbladder Polyps and Staging Gallbladder Cancer,” American Journal of Roentgenology 204, no. 2 (2015): W150–W159, 10.2214/AJR.13.11992. 25615775

[6] J. H. Choi , D. W. Seo , J. H. Choi , et al., “Utility of Contrast‐Enhanced Harmonic EUS in the Diagnosis of Malignant Gallbladder Polyps (With Videos),” Gastrointestinal Endoscopy 78, no. 3 (2013): 484–493, 10.1016/j.gie.2013.03.1328. 23642490

[7] H. Toyonaga , T. Hayashi , H. Ueki , et al., “An Intact Boundary Between the Tumor and Inner Hypoechoic Layer Discriminates T1 Lesions Among Sessile Elevated Gallbladder Cancers,” Journal of Hepato‐Biliary‐Pancreatic Sciences 28, no. 12 (2021): 1121–1129, 10.1002/jhbp.961. 33826798

[8] S. Hijioka , Y. Nagashio , A. Ohba , Y. Maruki , and T. Okusaka , “The Role of EUS and EUS‐FNA in Differentiating Benign and Malignant Gallbladder Lesions,” Diagnostics (Basel, Switzerland) 11, no. 9 (2021): 1586, 10.3390/diagnostics11091586. 34573929 PMC8467412

[9] J. H. Choi and D. W. Seo , “The Expanding Role of Contrast‐Enhanced Endoscopic Ultrasound in Pancreatobiliary Disease,” Gut and Liver 9, no. 6 (2015): 707–713, 10.5009/gnl15077. 26503571 PMC4625698

[10] H. P. Zhang , M. Bai , J. Y. Gu , Y. Q. He , X. H. Qiao , and L. F. Du , “Value of Contrast‐Enhanced Ultrasound in the Differential Diagnosis of Gallbladder Lesion,” World Journal of Gastroenterology 24, no. 6 (2018): 744–751, 10.3748/wjg.v24.i6.744. 29456413 PMC5807677

[11] T. Tamura , R. Ashida , and M. Kitano , “The Usefulness of Endoscopic Ultrasound in the Diagnosis of Gallbladder Lesions,” Frontiers in Medicine 9, no. 1 (2022): 957557, 10.3389/fmed.2022.957557. 36106323 PMC9465250

[12] B. J. Chang , S. H. Kim , H. Y. Park , et al., “Distinguishing Xanthogranulomatous Cholecystitis From the Wall‐Thickening Type of Early‐Stage Gallbladder Cancer,” Gut and Liver 4, no. 4 (2010): 518–523, 10.5009/gnl.2010.4.4.518. 21253302 PMC3021609

[13] M. Sugimoto , T. Takagi , N. Konno , et al., “The Efficacy of Contrast‐Enhanced Harmonic Endoscopic Ultrasonography in Diagnosing Gallbladder Cancer,” Scientific Reports 6 (2016): 25848, 10.1038/srep25848. 27162097 PMC4861928

[14] G. Leem , M. J. Chung , J. Y. Park , et al., “Clinical Value of Contrast‐Enhanced Harmonic Endoscopic Ultrasonography in the Differential Diagnosis of Pancreatic and Gallbladder Masses,” Clinical Endoscopy 51, no. 1 (2018): 80–88, 10.5946/ce.2017.044. 28928356 PMC5806916

[15] H. Imazu , N. Mori , K. Kanazawa , et al., “Contrast‐Enhanced Harmonic Endoscopic Ultrasonography in the Differential Diagnosis of Gallbladder Wall Thickening,” Digestive Diseases and Sciences 59, no. 8 (2014): 1909–1916, 10.1007/s10620-014-3115-5. 24664415

[16] N. Fujita , Y. Noda , G. Kobayashi , K. Kimura , and A. Yago , “Diagnosis of the Depth of Invasion of Gallbladder Carcinoma by EUS,” Gastrointestinal Endoscopy 50, no. 5 (1999): 659–663, 10.1016/S0016-5107(99)80015-7. 10536322

[17] M. Sugimoto , H. Irie , M. Takasumi , et al., “A Simple Method for Diagnosing Gallbladder Malignant Tumors With Subserosa Invasion by Endoscopic Ultrasonography,” BMC Cancer 21, no. 1 (2021): 288, 10.1186/s12885-021-08017-x. 33731052 PMC7972348

[18] K. Yoshida , T. Iwashita , S. Uemura , et al., “Efficacy of Contrast‐Enhanced EUS for Lymphadenopathy: A Prospective Multicenter Pilot Study (With Videos),” Gastrointestinal Endoscopy 90, no. 2 (2019): 242–250, 10.1016/j.gie.2019.03.015. 30922863

[19] X. Liang and X. Jing , “Retraction Note: Meta‐Analysis of Contrast‐Enhanced Ultrasound and Contrast‐Enhanced Harmonic Endoscopic Ultrasound for the Diagnosis of Gallbladder Malignancy,” BMC Medical Informatics and Decision Making 22, no. 1 (2022): 150, 10.1186/s12911-022-01895-6. 35672739 PMC9175489

[20] J. K. Park , Y. B. Yoon , Y.‐T. Kim , et al., “Management Strategies for Gallbladder Polyps: Is It Possible to Predict Malignant Gallbladder Polyps?,” Gut and Liver 2, no. 2 (2008): 88–94, 10.5009/gnl.2008.2.2.88. 20485616 PMC2871589

[21] N. R. Bhatt , A. Gillis , C. O. Smoothey , F. N. Awan , and P. F. Ridgway , “Evidence Based Management of Polyps of the Gall Bladder: A Systematic Review of the Risk Factors of Malignancy,” Surgeon 14, no. 5 (2016): 278–286, 10.1016/j.surge.2015.12.001. 26825588

[22] K. G. Foley , M. J. Lahaye , R. F. Thoeni , et al., “Management and Follow‐Up of Gallbladder Polyps: Updated Joint Guidelines Between the ESGAR, EAES, EFISDS and ESGE,” European Radiology 32, no. 5 (2022): 3358–3368, 10.1007/s00330-021-08384-w. 34918177 PMC9038818

[23] M. Kitano , Y. Yamashita , K. Kamata , et al., “The Asian Federation of Societies for Ultrasound in Medicine and Biology (AFSUMB) Guidelines for Contrast‐Enhanced Endoscopic Ultrasound,” Ultrasound in Medicine & Biology 47, no. 6 (2021): 1433–1447, 10.1016/j.ultrasmedbio.2021.01.030. 33653627

[24] K. Numata , H. Oka , M. Morimoto , et al., “Differential Diagnosis of Gallbladder Diseases With Contrast‐Enhanced Harmonic Gray Scale Ultrasonography,” Journal of Ultrasound in Medicine 26, no. 6 (2007): 763–774, 10.7863/jum.2007.26.6.763. 17526608

[25] H.‐X. Yuan , W.‐P. Wang , J.‐X. Wen , Z.‐B. Ji , H. Ding , and B.‐J. H. C.‐L. Li , “Xanthogranulomatous Cholecystitis: Contrast‐Enhanced Ultrasound Features and Differential Diagnosis From Wall‐Thickening Gallbladder Carcinoma,” Discovery Medicine 21, no. 114 (2016): 89–98. 27011044

[26] M. Kitano , K. Kamata , H. Imai , et al., “Contrast‐Enhanced Harmonic Endoscopic Ultrasonography for Pancreatobiliary Diseases,” Digestive Endoscopy 27 (2015): 60–67, 10.1111/den.12454. 25639788

