# Supplementary material for: Efficacy of Contrast‐Enhanced Endoscopic Ultrasound in the Diagnosis of Gallbladder Tumor: A Retrospective Multicenter Cohort Study
Source: J Hepatobiliary Pancreat Sci. 2026 Jan 23;33(5):380–8. doi: 10.1002/jhbp.70069 (PMC13206424; doi:10.1002/jhbp.70069)
Supplement: Supplementary file 1 — Table S1: Wall layer structure and enhancement pattern of the benign and malignant gallbladder nodules in CE‐EUS. Table S2: Wall layer structures and enhancement patterns on CE‐EUS according to depth of invasion in gallbladder cancer. Table S3: Interobserver agreement for B‐mode EUS and CE‐EUS findings in gallbladder nodules. [file JHBP-33-380-s002.docx]

**Supplementary** **Table 1.** **Wall layer structure and enhancement pattern of the benign and malignant gallbladder nodules in CE-EUS.**

|  | **CE-EUS image analysis** | | | | | |
| --- | --- | --- | --- | --- | --- | --- |
|  | **Wall layer structure** | | | **Enhancement pattern** | | |
|  | **clear** | **unclear** | **disrupted** | **homogeneous** | **heterogeneous** | **non** |
| Malignant , n (%) | 4 (13) | 21 (68) | 6 (19) | 13 (42) | 18 (58) | 0 (0) |
| Benign, n (%) | 52 (95) | 3 (5) | 0 (0) | 44 (80) | 1 (2) | 10 (18) |
| -Adenomyomatosis, n (%) | 16 (31) | 1 (33) | 0 (0) | 17 (39) | 0 (0) | 0 (0) |
| -Chronic cholecystitis, n (%) | 13 (25) | 0 (0) | 0 (0) | 13 (30) | 0 (0) | 0 (0) |
| -Biliary sludge, n (%) | 10 (19) | 0 (0) | 0 (0) | 0 (0) | 0 (0) | 10 (100) |
| -Cholesterol polyp, n (%) | 8 (15) | 0 (0) | 0 (0) | 8 (18) | 0 (0) | 0 (0) |
| -Inflammatory polyp, n (%) | 2 (4) | 0 (0) | 0 (0) | 2 (5) | 0 (0) | 0 (0) |
| -Adenoma, n (%) | 3 (6) | 0 (0) | 0 (0) | 3 (7) | 0 (0) | 0 (0) |
| -Xanthogranulomatous cholecystitis, n (%) | 0 (0) | 2 (67) | 0(0) | 1 (2) | 1 (100) | 0 (0) |

CE-EUS, contrast-enhanced Endoscopic Ultrasonography.

**Supplementary Table 2. Wall layer structures and enhancement patterns on CE-EUS according to depth of invasion in gallbladder cancer.**

|  | **CE-EUS image analysis** | | | | | |
| --- | --- | --- | --- | --- | --- | --- |
|  | **Wall layer structure** | | | **Enhancement pattern** | | |
|  | **clear** | **unclear** | **disrupted** | **homogeneous** | **heterogeneous** | **non** |
| Tis , n | 2 | 0 | 0 | 2 | 0 | 0 |
| T1a, n | 2 | 0 | 0 | 2 | 0 | 0 |
| T1b, n | 0 | 1 | 0 | 1 | 0 | 0 |
| T2a, n | 0 | 13 | 0 | 7 | 6 | 0 |
| T2b, n | 0 | 7 | 0 | 1 | 6 | 0 |
| T3, n | 0 | 0 | 6 | 0 | 6 | 0 |

CE-EUS, contrast-enhanced Endoscopic Ultrasonography.

**Supplementary Table 3. Interobserver agreement for B-mode EUS and CE-EUS findings in gallbladder nodules.**

| **Modality** | **Variable** | **κ (95 %CI)** | **Interpretation** |
| --- | --- | --- | --- |
| B-mode EUS | wall layer structure | 0.77 (0.62-0.91) | Substantial |
|  | RAS | 0.79 (0.66-0.92) | Substantial |
|  | echo texture | 0.72 (0.58-0.86) | Substantial |
|  | echo genicity | 0.75 (0.61-0.89) | Substantial |
|  | shape | 0.85 (0.75-0.97) | Almost perfect |
| CE-EUS | enhancement pattern | 0.81 (0.65-0.93) | Almost perfect |
|  | wall layer structure | 0.79 (0.60-0.99) | Substantial |

EUS, endoscopic ultrasonography; CE-EUS, contrast-enhanced endoscopic ultrasonography; RAS, Rokitansky-Aschoff sinus.
