# Supplementary material for: Efficacy of Contrast‐Enhanced Endoscopic Ultrasound in the Diagnosis of Gallbladder Tumor: A Retrospective Multicenter Cohort Study
Source: J Hepatobiliary Pancreat Sci. 2026 Jan 23;33(5):380–8. doi: 10.1002/jhbp.70069 (PMC13206424; doi:10.1002/jhbp.70069)
Supplement: Supplementary file 2 — Figure S1: Typical B‐mode EUS images demonstrating gallbladder wall layer structure: (a) a benign gallbladder nodule (cholesterol polyp) with a clear internal hypoechoic layer, (b) a malignant gallbladder nodule (adenocarcinoma) with an unclear internal hypoechoic layer, and (c) a malignant gallbladder nodule (adenocarcinoma) with disruption of the outer hyperechoic layer. EUS, endoscopic ultrasonography. Figure S2: Typical CE‐EUS images demonstrating gallbladder wall layer structure: (a) a benign gallbladder nodule (cholesterol polyp) with a clear internal hypoechoic layer, (b) a malignant gallbladder nodule (adenocarcinoma) with an unclear inner hypoechoic layer, and (c) a malignant gallbladder nodule (adenocarcinoma) with disruption of the outer hyperechoic layer. CE‐EUS, contrast‐enhanced endoscopic ultrasonography. Figure S3: Typical CE‐EUS images demonstrating enhancement patterns of gallbladder nodules: (a) a benign gallbladder nodule (adenoma) with homogeneous enhancement, (b) a malignant gallbladder nodule (adenocarcinoma) with heterogeneous enhancement, and (c) a benign gallbladder nodule (gallbladder sludge) showing no enhancement. CE‐EUS, contrast‐enhanced endoscopic ultrasonography. Figure S4: Flowchart of study enrollment. CE‐EUS, contrast‐enhanced endoscopic ultrasonography. Figure S5: Receiver operating characteristic (ROC) curve of the maximum diameter measured by B‐mode EUS for differentiating benign from malignant gallbladder nodules. ROC, Receiver operating characteristic; EUS, endoscopic ultrasonography. Figure S6: ROC curves comparing B‐mode EUS and CE‐EUS wall layer structure for differentiating benign from malignant gallbladder nodules (AUC 0.77 vs. 0.91) ROC, Receiver operating characteristic; EUS, endoscopic ultrasonography; CE‐EUS, contrast‐enhanced endoscopic ultrasonography. [file JHBP-33-380-s001.zip › jhbp70069-sup-0007-FigureS1-S6@Supplementary Figure legends.docx]

**Supplementary Figure legends**

Supplementary Figure 1.:

Typical B-mode EUS images demonstrating gallbladder wall layer structure: (a) a benign gallbladder nodule (cholesterol polyp) with a clear internal hypoechoic layer, (b) a malignant gallbladder nodule (adenocarcinoma) with an unclear internal hypoechoic layer, and (c) a malignant gallbladder nodule (adenocarcinoma) with disruption of the outer hyperechoic layer.

EUS, endoscopic ultrasonography.

Supplementary Figure 2.:

Typical CE-EUS images demonstrating gallbladder wall layer structure: (a) a benign gallbladder nodule (cholesterol polyp) with a clear internal hypoechoic layer, (b) a malignant gallbladder nodule (adenocarcinoma) with an unclear inner hypoechoic layer, and (c) a malignant gallbladder nodule (adenocarcinoma) with disruption of the outer hyperechoic layer.

CE-EUS, contrast-enhanced endoscopic ultrasonography.

Supplementary Figure 3.:

Typical CE-EUS images demonstrating enhancement patterns of gallbladder nodules: (a) a benign gallbladder nodule (adenoma) with homogeneous enhancement, (b) a malignant gallbladder nodule (adenocarcinoma) with heterogeneous enhancement, and (c) a benign gallbladder nodule (gallbladder sludge) showing no enhancement.

CE-EUS, contrast-enhanced endoscopic ultrasonography.

Supplementary Figure 4.:

Flowchart of study enrollment.

CE-EUS, contrast-enhanced endoscopic ultrasonography.

Supplementary Figure 5.:

Receiver operating characteristic (ROC) curve of the maximum diameter measured by B-mode EUS for differentiating benign from malignant gallbladder nodules.

ROC, Receiver operating characteristic; EUS, endoscopic ultrasonography.

Supplementary Figure 6.:

ROC curves comparing B-mode EUS and CE-EUS wall layer structure for differentiating benign from malignant gallbladder nodules (AUC 0.77 vs 0.91)

ROC, Receiver operating characteristic; EUS, endoscopic ultrasonography; CE-EUS, contrast-enhanced endoscopic ultrasonography.
